# A YOLOv5-based network for the detection of a diffuse reflectance spectroscopy probe to aid surgical guidance in gastrointestinal cancer surgery

**DOI:** 10.1007/s11548-023-02944-9

**Published:** 2023-06-08

**Authors:** Ioannis Gkouzionis, Yican Zhong, Scarlet Nazarian, Ara Darzi, Nisha Patel, Christopher J. Peters, Daniel S. Elson

**Affiliations:** 1 Hamlyn Center, Imperial College London, London, UK; 2 Department of Surgery and Cancer, Imperial College London, London, UK

**Keywords:** YOLOv5, Deep learning, Probe detection, Spectroscopy, Cancer surgery

## Abstract

**Purpose:**

A positive circumferential resection margin (CRM) for oesophageal and gastric carcinoma is associated with local recurrence and poorer long-term survival. Diffuse reflectance spectroscopy (DRS) is a non-invasive technology able to distinguish tissue type based on spectral data. The aim of this study was to develop a deep learning-based method for DRS probe detection and tracking to aid classification of tumour and non-tumour gastrointestinal (GI) tissue in real time.

**Methods:**

Data collected from both ex vivo human tissue specimen and sold tissue phantoms were used for the training and retrospective validation of the developed neural network framework. Specifically, a neural network based on the You Only Look Once (YOLO) v5 network was developed to accurately detect and track the tip of the DRS probe on video data acquired during an ex vivo clinical study.

**Results:**

Different metrics were used to analyse the performance of the proposed probe detection and tracking framework, such as precision, recall, mAP 0.5, and Euclidean distance. Overall, the developed framework achieved a 93% precision at 23 FPS for probe detection, while the average Euclidean distance error was 4.90 pixels.

**Conclusion:**

The use of a deep learning approach for markerless DRS probe detection and tracking system could pave the way for real-time classification of GI tissue to aid margin assessment in cancer resection surgery and has potential to be applied in routine surgical practice.

## Introduction

Cancers of the gastrointestinal (GI) tract remain a major contributor to the global cancer risk. The aim of surgery is for complete resection of tumour with clear margins, while preserving as much surrounding healthy tissue as possible [1]. A positive circumferential resection margin (CRM) is associated with local recurrence of the tumour and poorer long-term survival. The accurate mapping of tumour margins is of particular importance for curative cancer resection and improvement in overall survival. Current mapping techniques preclude a full resection margin assessment in real time.

**Fig. 1:**
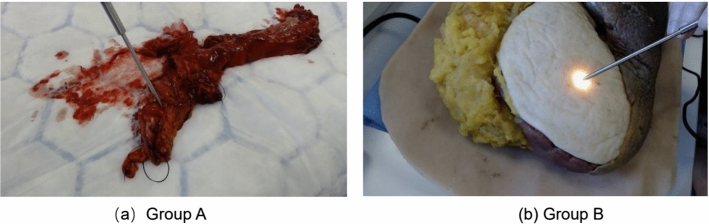
Video data from human excised tissue “Group A” (left) and tissue phantom “Group B” (right)

Currently, the gold-standard intra-operative technique for CRM assessment is frozen sections [2]. However, this technique is at risk of sampling errors, plus it is time-consuming, labour- intensive, and lengthens the operative time, affecting both patient outcome and theatre efficiency [3]. These challenges can potentially be addressed by using multispectral optical probes, which have been previously shown to have high sensitivity and specificity (greater than 90%) for discriminating between normal and cancer tissue [4].

Diffuse reflectance spectroscopy (DRS), a point-based spectroscopy technique, allows discrimination of normal and abnormal tissue based on spectral data and presents a promising advancement in cancer diagnosis [5]. The main limitation of the clinical use of DRS is that although DRS can discriminate tissue types, it does so by providing single-point spectral measurements and leaves no marks on the tissue during scanning [6]. In this way, it is not possible to localise the area that has been in contact with the probe when optical biopsy takes place, and thus makes it difficult for the surgeon to determine the resection margin. This is particularly challenging when DRS is used endoscopically or during minimally invasive surgery (MIS), where the ergonomics of scanning and viewing the DRS probe site are even more demanding. To overcome this limitation and localise the optical biopsy sites on the specimen, an optical tracking method was developed, as described in previous work [7]. Briefly, to track the DRS probe, a colour marker was chosen based on the colour distribution of biological tissue in the hue saturation value (HSV) colour space. A green colour marker was wrapped around the distal end of the DRS probe to allow detection of the probe. Tracking of the probe was achieved using a Kalman filter. The exact probe coordinates at each sampling point were recorded. In this way, the localisation of the probe tip was known in real time.

The main limitation of this approach was that a marker is required to be attached on the probe’s shaft. This is challenging when it comes to the in vivo clinical setting, as a biocompatible sterilised marker is needed to be attached to the probe, a process that highly interferes with the surgical workflow. Additionally, the colour marker is prone to occlusion from blood that leads to inaccurate detection of the probe during the surgical operation. To overcome this limitation and localise the optical biopsy sites on the specimen, this paper presents a novel deep learning-based detection and tracking system to enable markerless real-time localisation of the tip of the handheld DRS probe. The system allowed tracking of the two-dimensional (2D) position and orientation of the DRS probe using image data (Fig. 1).

## Methodology

The aim of this study was to develop a robust deep learning framework for accurate detection and tracking of the tip of the DRS probe. Two video datasets were used for the training and testing of our deep learning framework. The first dataset, Group A, comprised of ex vivo video data acquired at Imperial NHS Trust (ref. no. 08/H0719/37). More specifically, once the human tissue specimen was excised from the patient, a video of the DRS probe sampling the tissue was taken. For the second dataset, Group B, a solid tissue phantom was used for the collection of the video data. In total, 11 videos were acquired for both Groups A and B at 1920 $\times$ 1080 resolution and 30 frames per second (FPS), while the length of the videos ranged from 60 to 120 s.

**Fig. 2:**
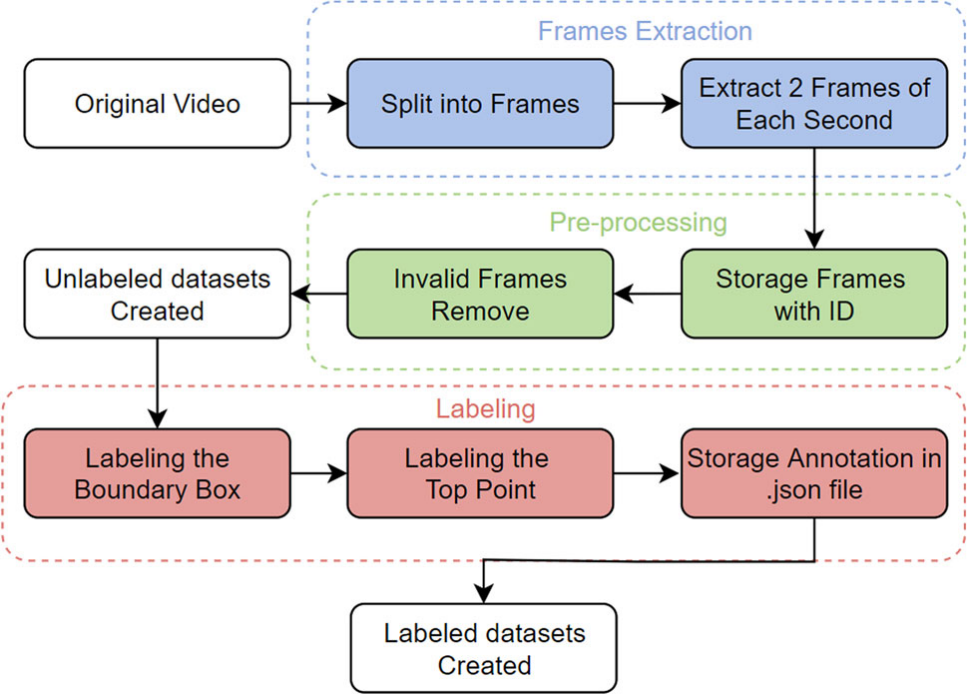
Video data processing and labelling pipeline

Following the processing pipeline shown in Fig. 2, we ended up with a total of 1942 frames that were then labelled using the open-source labelling tool Labelbox (https://labelbox.com). Two annotation types were used, namely the bounding box around the metal shaft of the probe and the tip point of the DRS probe. To address the requirements of deep learning methods for big data, the video dataset was further increased using image augmentation techniques. Specifically, the Mosaic [8], mix-Up [9], and non-max suppression [10] methods were used.

**Fig. 3:**
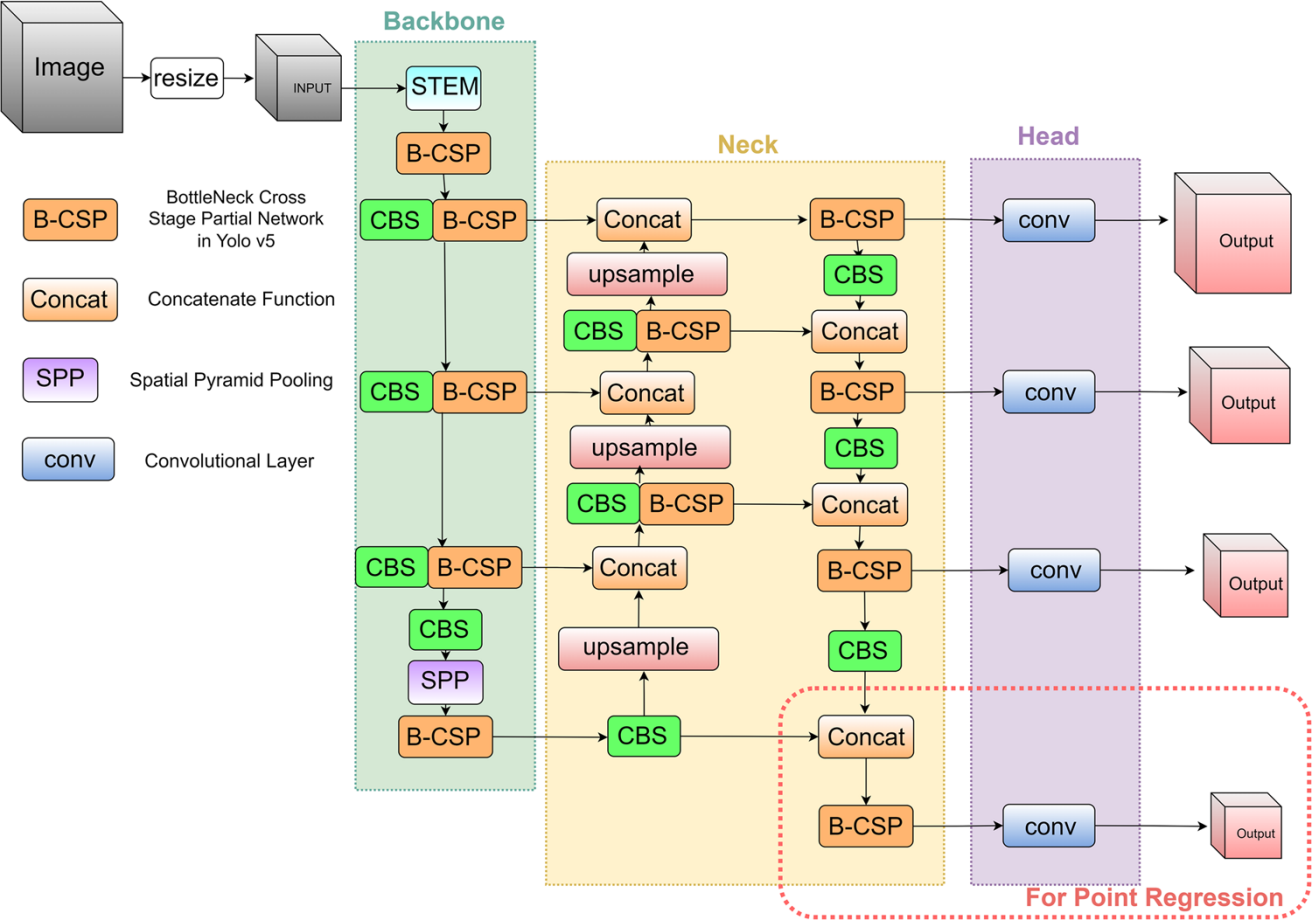
Developed YOLOv5-based network architecture with the STEM layer

**Fig. 4:**
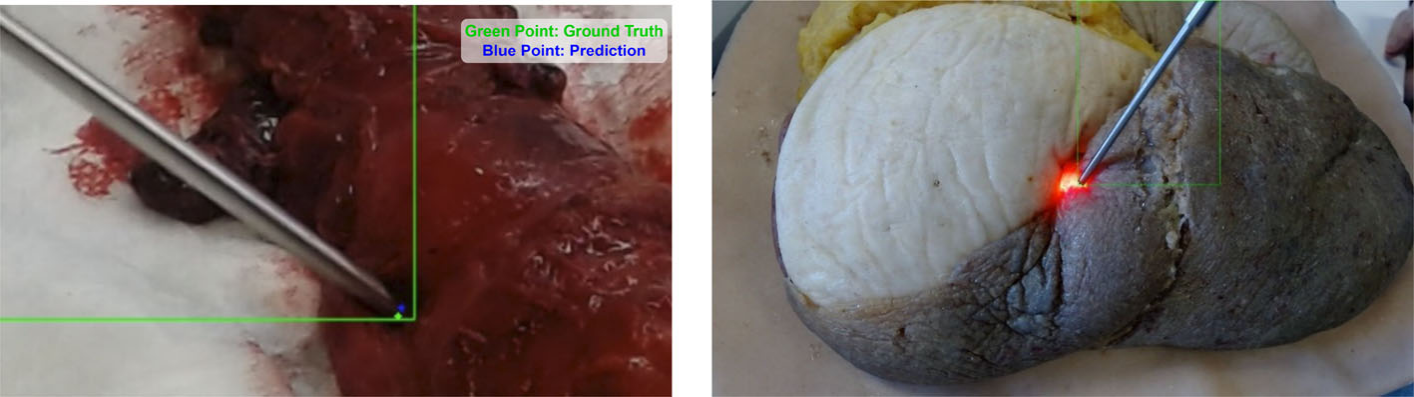
DRS probe tip point detection. The green point is ground truth, and the blue point is the prediction by our model

The developed model was based on the implementation of You Only Look Once (YOLO) v5 network [10]. YOLO formulates the object detection task as a unified, end-to-end regression problem resulting in a fast and generalisable framework. Compared to YOLO v5, the network modifies the CBL block by replacing the Leaky ReLU activation function with the SiLU activation function. The detailed structure update of the network is shown in Fig. 3. The YOLO v5’s Focus Layer is replaced by the STEM layer, which is considered to improve the generalisation ability of the network reducing at the same time its computational complexity. For the DRS probe tip detection, a four-point tip regression was also added to the network to minimise probe detection errors.

## Results

To measure the performance of the developed deep learning model, precision, recall, mAP0.5, and mAP0.5:0.95 were employed. For point tracking, the Euclidean distance between the predicted and ground truth tip points was calculated at pixel level. For the training of the developed network, data were split into training, validation, and testing using a ratio of 8:1:1. The model was trained on a NVIDIA 2080 Ti GPU-powered machine. In total, 760 epochs were used leading to a detection precision of 0.76, mAP0.5 of 0.99, and mAP0.5:0.95 of 0.88, while the average Euclidean error was 7.13 pixels. Furthermore, the average error for the Group A was significantly lower compared to that of Group B, as 85.22% of the error value is less than 10 pixels. Overall, an average error of 4.90 pixels and a prediction precision of 93.67% were achieved (Fig. 4). In the inference mode, the developed model was able to detect and track the DRS probe in 23 FPS.

## Discussion and conclusion

In this study, a deep learning framework for the DRS probe detection was developed to support clinicians with the complete tumour detection and resection. The network is able to detect and track the tip of the probe with 93% accuracy in near real time at 23 FPS. The real-time probe detection and tracking method developed in this study can also be applied to other optical spectroscopy techniques, such as rapid evaporative ionisation mass spectrometry (REIMS) technology, fluorescence spectroscopy, and Raman spectroscopy. In this way, the ergonomics, ease of use, and validation of data collection for these optical techniques can be improved.

The proposed deep learning-based DRS probe detection and tracking network has been validated on ex vivo data, and the accuracy derived demonstrates the strength and clinical value of the technique. The method allows real-time probe tracking and could aid resection margin assessment in cancer surgery and has potential to be applied in routine surgical practice.
